# Leukocyte telomere length as a diagnostic biomarker for anti-tuberculosis drug-induced liver injury

**DOI:** 10.1038/s41598-020-62635-2

**Published:** 2020-03-27

**Authors:** Wanvisa Udomsinprasert, Noppadol Chanhom, Supharat Suvichapanich, Sukanya Wattanapokayakit, Surakameth Mahasirimongkol, Wasun Chantratita, Jiraphun Jittikoon

**Affiliations:** 1 Department of Biochemistry, Faculty of Pharmacy, Mahidol University, Bangkok, 10400 Thailand; 2 Genomic Medicine Centre, Division of Genomic Medicine and Innovation Support, Department of Medical Sciences, Ministry of Public Health, Nonthaburi, 11000 Thailand; 3 Center for Medical Genomics, Faculty of Medicine, Ramathibodi Hospital, Mahidol University, Bangkok, 10400 Thailand

**Keywords:** Biomarkers, Translational research

## Abstract

Despite being relatively rare, anti-tuberculosis drug-induced liver injury (ATDILI) is a leading cause of acute liver failure and a major reason for treatment discontinuation, because of no specific and selective markers for ATDILI. Herein, this study aimed to investigate whether telomere length, a biological indicator of age-related diseases, is associated with ATDILI outcomes and could serve as an early ATDILI biomarker. Relative telomere length (RTL) in blood leukocyte of 100 age- and gender-matched healthy controls, 49 tuberculosis patients with ATDILI, and 53 tuberculosis patients with non-ATDILI was quantified using real-time polymerase chain reaction. Both tuberculosis patients with and without ATDILI had significantly shorter RTL than healthy controls. Compared with tuberculosis patients with non-ATDILI, RTL in those with ATDILI was significantly increased. Longer RTL was found to be significantly associated with increased susceptibility to ATDILI. Multivariate linear regression analysis showed that an increment in RTL was independently correlated with elevated values of aspartate aminotransferase and alanine aminotransferase assessed within 60 days after anti-tuberculosis treatment. Kaplan-Meier curve analysis demonstrated that longer RTL was associated with elevated rates of hepatotoxicity in tuberculosis patients. Receiver-operating characteristic curve analysis unveiled a diagnostic accuracy of RTL as a novel indicator for ATDILI progression (AUC = 0.73), which yielded more sensitive and specific values than traditional liver biomarkers including serum enzyme activities of aminotransferases measured within 7 days after treatment with anti-tuberculosis regimens. Collectively, aberrant RTL in blood leukocyte would reflect hepatotoxicity induced by anti-tuberculosis agents and might have a potential biomarker for early ATDILI progression.

## Introduction

Tuberculosis is a chronic respiratory infectious disease and a leading cause of morbidity and mortality worldwide. The disease is characterized by *Mycobacterium tuberculosis* (MTB) infection usually affecting the lungs, leading to severe coughing, fever, and chest pains^1^ that mimic features of other pathologies, thereby becoming a major public health problem. Currently, standard treatment regimens for tuberculosis patients consist of isoniazid, rifampicin, pyrazinamide, and ethambutol that shorten the treatment period and enhance the therapeutic efficacy^2^. However, these anti-tuberculosis agents have been reported to be the most important causative drug-induced liver injury (DILI) in much of developing world^3^, ultimately leading to irreversible liver failure. Despite refined usage of traditional liver biomarkers including serum enzyme activities of aminotransferases and bilirubin levels^4^, these biomarkers are inadequate for early identification of DILI, and alterations in those conventional tools are not mechanistically informative. For these reasons, it would seem more appropriate to identify and develop new more sensitive and specific DILI biomarkers, which may be helpful in improving early DILI prediction and subsequent patient outcomes. It is imperative to better understand the exact causes of hepatotoxicity in tuberculosis patients, which could lead to not only the discovery of potential biomarkers for predicting DILI at an earlier stage than the currently used indicators, but also the identification of treatment responses in tuberculosis patients.

Given that age has been considered to be one of risk factors for anti-tuberculosis drug-induced liver injury (ATDILI)^5,6^, telomeres recognized as biological indicators of age-related diseases may have an immense potential to be diagnostic biomarkers for the developmental and progressive ATDILI. As repetitive DNA sequences of TTAGGG and an associated protein complex at chromosome ends, telomeres are essential for chromosome end protection (telomere capping) and chromosomal stability^7^. In general, telomere length shortens each time cells divide, because DNA polymerases are not capable of completely replicating chromosomes during cell division. Under pathological conditions, alterations in telomere length precipitate loss of capping function at the chromosomal ends, which in turn alter DNA damage program contributing to cellular senescence, apoptosis, and neoplastic transformation^8^. On the basis of its property, shortened telomeres have been reportedly associated with a vast number of age-related diseases, particularly pulmonary diseases including pulmonary fibrosis^9^ and chronic obstructive pulmonary disease (COPD)^10^. In addition to chronic lung diseases, telomere shortening has been evinced to drive the progression of liver cirrhosis in both hepatocytes and senescence associated with fibrotic scaring^8^. Apart from telomeres in the liver-specific cells, several clinical studies uncovered telomere attrition in blood leukocytes of patients with chronic liver diseases^10–15^. Collectively, it seems plausible that aberrant telomere length could be an important mediator for hepatocyte damage and turnover, and alterations in telomere length would open a unique opportunity for early detection of ATDILI progression in tuberculosis patients.

Although telomere length in age-related pulmonary diseases and chronic liver conditions has been extensively explored, until now no attempt has been made to capture the breadth of telomere length related to ATDILI progression. Accordingly, the present study aimed to investigate telomere length in blood leukocyte of tuberculosis patients with and without ATDILI compared with age- and gender-matched healthy volunteers. Whether telomere length in blood leukocyte is associated with liver function parameters and could be utilized as a possible biomarker identifying the development and progression of ATDILI in tuberculosis patients was further determined.

## Results

### Demographic and clinical characteristics of tuberculosis patients

Baseline demographic and clinical characteristics of tuberculosis patients with and without ATDILI are summarized in Table 1. Mean age, gender ratio, and body mass index (BMI) in healthy controls (47.25 ± 15.78 years, 31 women and 69 men, 19.42 ± 3.72 kg/m^2^) and tuberculosis patients (48.22 ± 16.05 years, 37 women and 65 men, 18.79 ± 2.75 kg/m^2^) were not significantly different. In regard to ATDILI status of tuberculosis patients, there were no significant differences in age, gender ratio, BMI, and liver function parameters including aspartate aminotransferase (AST), alanine aminotransferase (ALT), alkaline phosphatase (ALP), total bilirubin (TB), and direct bilirubin (DB) measured within 7 days after commencement of anti-tuberculosis treatment between the patients with ATDILI and non-ATDILI. As expected, tuberculosis patients with ATDILI showed substantially higher AST, ALT, TB, and DB values assessed within 60 days during therapy than those without ATDILI (*P* < 0.001, *P* < 0.001, *P* = 0.02, *P* = 0.02, respectively). However, the values of AST and ALT measured within 60 days after treatment in the patients with ATDILI showed a high variation, which ranged from 34 to 1,068 IU/L and 13 to 488 IU/L, respectively.

**Table 1:** Clinical characteristics of tuberculosis patients with and without ATDILI assessed within 7 days and 60 days after commencement of anti-tuberculosis treatment.

Variables	Within 7 days after treatment		*P* value	Within 60 days after treatment		*P* value
	ATDILI	Non-ATDILI		ATDILI	Non-ATDILI	
Number	49	53		49	53	
Age (years)	51.11 ± 16.40	45.45 ± 15.36	0.09	51.11 ± 16.40	45.45 ± 15.36	0.09
Gender (F/M)	21 (42.9%)/28 (57.1%)	16 (30.2%)/37 (69.8%)	0.22	21 (42.9%)/28 (57.1%)	16 (30.2%)/37 (69.8%)	0.22
BMI (kg/m^2^)	19.18 ± 4.72	19.62 ± 2.91	0.67	19.18 ± 4.72	19.62 ± 2.91	0.67
AST (IU/L)	47.08 ± 88.71	34.80 ± 16.03	0.60	232.79 ± 209.13	27.88 ± 12.44	<0.001
ALT (IU/L)	55.19 ± 130.02	31.53 ± 22.31	0.49	134.78 ± 99.54	26.57 ± 16.48	<0.001
ALP (IU/L)	131.90 ± 113.74	96.77 ± 58.03	0.31	176.39 ± 139.97	115.31 ± 81.35	0.20
TB (mg/dL)	1.10 ± 1.19	0.46 ± 0.30	0.08	2.44 ± 3.50	0.63 ± 0.34	0.02
DB (mg/dL)	0.48 ± 0.80	0.15 ± 0.04	0.51	1.57 ± 2.33	0.28 ± 0.25	0.02

Abbreviations: ALP, alkaline phosphatase; ALT, alanine aminotransferase; AST, aspartate aminotransferase; ATDILI, anti-tuberculosis drug-induced liver injury; BMI, body mass index; DB, direct bilirubin; F, female; M, male; TB, total bilirubin.

To further identify risk factors associated with ATDILI in tuberculosis patients, we performed a univariate logistic regression analysis. The analysis revealed no significant associations of age, gender, BMI, drinking status, and smoking status with susceptibility to ATDILI in tuberculosis patients (Supplementary Table 1).

### Increased RTL in tuberculosis patients with ATDILI

**Figure 1:**
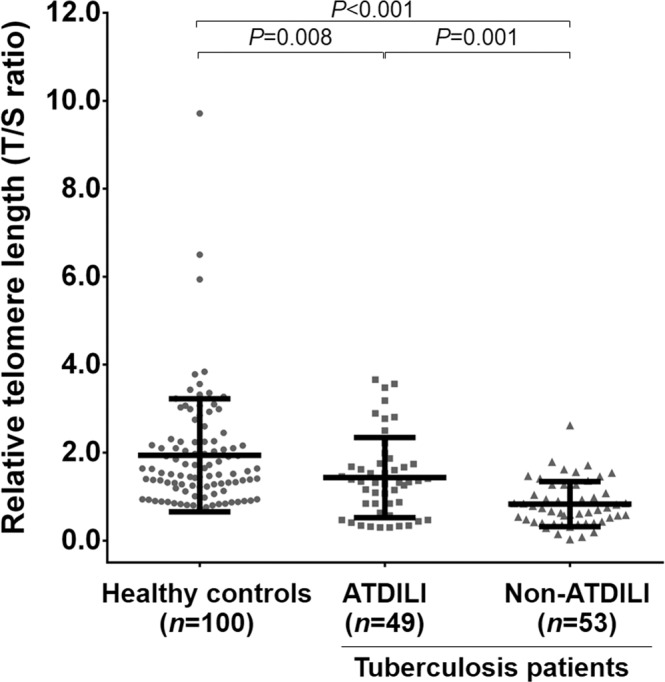
RTL in blood leukocyte of healthy controls and tuberculosis patients with and without ATDILI. Abbreviations: ATDILI, anti-tuberculosis drug-induced liver injury; RTL, relative telomere length.

We firstly investigated telomere length in blood leukocyte from tuberculosis patients with and without ATDILI, in addition to unaffected controls. As depicted in Fig. 1, compared with age- and gender-matched healthy controls, tuberculosis patients with ATDILI had remarkably reduced RTL (*P* = 0.008). Likewise, the patients without ATDILI remained significantly shorter RTL than the controls (*P* < 0.001). Instead, RTL in blood leukocytes of tuberculosis patients with ATDILI was substantially higher than that of the patients without ATDILI (*P* = 0.001).

### Long RTL as an independent risk factor for ATDILI

Owing to elevated RTL in tuberculosis patients with ATDILI compared with those without ATDILI, we performed unconditional logistic regression analysis to examine an association between RTL and ATDILI risk. Using the median distribution of RTL in healthy controls, RTL in blood leukocyte of tuberculosis patients was categorized into longer RTL (RTL > 1.31, *n* = 32) and shorter RTL (RTL ≤ 1.31, *n* = 70). As shown in Table 2, tuberculosis patients with longer RTL exhibited significantly an elevated risk of ATDILI, compared with those with shorter RTL in both univariate (unadjusted odds ratio, OR: 10.35, 95% confident interval, CI: 3.53 to 30.30, *P* < 0.001) and multivariate analyses (adjusted OR: 26.38, 95% CI: 5.14 to 135.55, *P* < 0.001). We further classified the patients into three groups, based on the tertile of RTL values in unaffected controls and revealed a significant dose-response relationship between longer RTL and an augmented risk of ATDILI in tuberculosis patients. Specifically, using the third tertile (shortest RTL) as the reference group, the OR values for the first and second tertiles were 4.47 (95% CI: 1.82 to 10.98, *P* = 0.001) and 3.61 (95% CI: 1.16 to 11.22, *P* = 0.027), respectively, in an unadjusted univariate model and 6.46 (95% CI: 2.10 to 19.86, *P* = 0.001) and 5.17 (95% CI: 2.05 to 25.52, *P* = 0.014), respectively, in an adjusted multivariate model.

**Table 2:** Associations between blood leukocyte RTL and risk of ATDILI.

RTL	Tuberculosis patients		Unadjusted	*P*-value	Adjusted	*P*-value
	ATDILI	Non-ATDILI	OR (95% CI)		OR (95% CI)	
Overall	49	53	3.51 (1.73 to 7.12)	**0.001**	7.27 (2.31 to 22.86)	**0.001**
**By median**						
Longer	26 (53.1%)	6 (11.3%)	10.35 (3.53 to 30.30)	**< 0.001**	26.38 (5.14 to 135.55)	**< 0.001**
Shorter	23 (46.9%)	47 (88.7%)	1		1	
**By tertile**						
1^st^ tertile	16 (32.7%)	2 (3.7%)	4.47 (1.82 to 10.98)	**0.001**	6.46 (2.10 to 19.86)	**0.001**
2^nd^ tertile	23 (46.9%)	37 (69.8%)	3.61 (1.16 to 11.22)	**0.027**	5.17 (2.05 to 25.52)	**0.014**
3^rd^ tertile	10 (20.4%)	12 (22.6%)	1		1	

Adjusted for age, gender, BMI, drinking status, and smoking status.

Abbreviations: ATDILI, anti-tuberculosis drug-induced liver injury; BMI, body mass index; CI, confident interval; OR, odds ratio; RTL, relative telomere length.

### Relationships between RTL and clinical parameters indicating hepatotoxicity

We subsequently determined whether RTL in blood leukocyte is associated with clinical variables of ATDILI progression in tuberculosis patients. The relationship between RTL and liver function parameters measured within 60 days during therapy is demonstrated in Table 3. Spearman’s rho correlation analysis disclosed the significantly positive correlations of blood leukocyte RTL with AST (*r* = 0.51, *P* < 0.001), ALT (*r* = 0.51, *P* < 0.001), TB (*r* = 0.44, *P* = 0.020), and DB (*r* = 0.46, *P* = 0.011) in both tuberculosis patients with and without ATDILI. To further verify the independent correlations of RTL with the aforementioned parameters, we conducted multivariate linear regression analysis. After adjustments for confounding variables including age, gender, BMI, drinking status, and smoking status, an increment in RTL was found to be independently associated with raised values of AST (β-coefficient = 0.003; 95% CI: 0.002 to 0.004; *P* < 0.001) and ALT (β-coefficient = 0.007; 95% CI: 0.004 to 0.009; *P*  < 0.001) (Table 3).

**Table 3:** Spearman’s rho correlation and multivariate linear regression analyses of RTL estimates.

Variables	Relative telomere length (T/S ratio)			
	Spearman’s rho correlation		Linear regression^a^	
	Coefficient (*r*)	*P*-value	β coefficient (95% CI)	*P*-value
Age (years)	0.53	0.63	—	—
Gender (F/M)	−0.27	0.79	—	—
BMI (kg/m^2^)	−1.74	0.21	—	—
AST (IU/L)	0.51	**< 0.001**	0.003 (0.002 to 0.004)	**< 0.001**
ALT (IU/L)	0.51	**< 0.001**	0.007 (0.004 to 0.009)	**< 0.001**
ALP (IU/L)	0.25	0.14	—	—
TB (mg/dL)	0.44	**0.020**	−0.27 (−0.16 to 0.11)	0.69
DB (mg/dL)	0.46	**0.011**	−0.47 (−0.28 to 0.18)	0.66

Adjusted for age, gender, BMI, drinking status, and smoking status.

Abbreviations: ALP, alkaline phosphatase; ALT, alanine aminotransferase; AST, aspartate aminotransferase; ATDILI, anti-tuberculosis drug-induced liver injury; BMI, body mass index; DB, direct bilirubin; F, female; M, male; TB, total bilirubin.

### Increased rates of ATDILI development in tuberculosis patients with longer RTL

**Figure 2:**
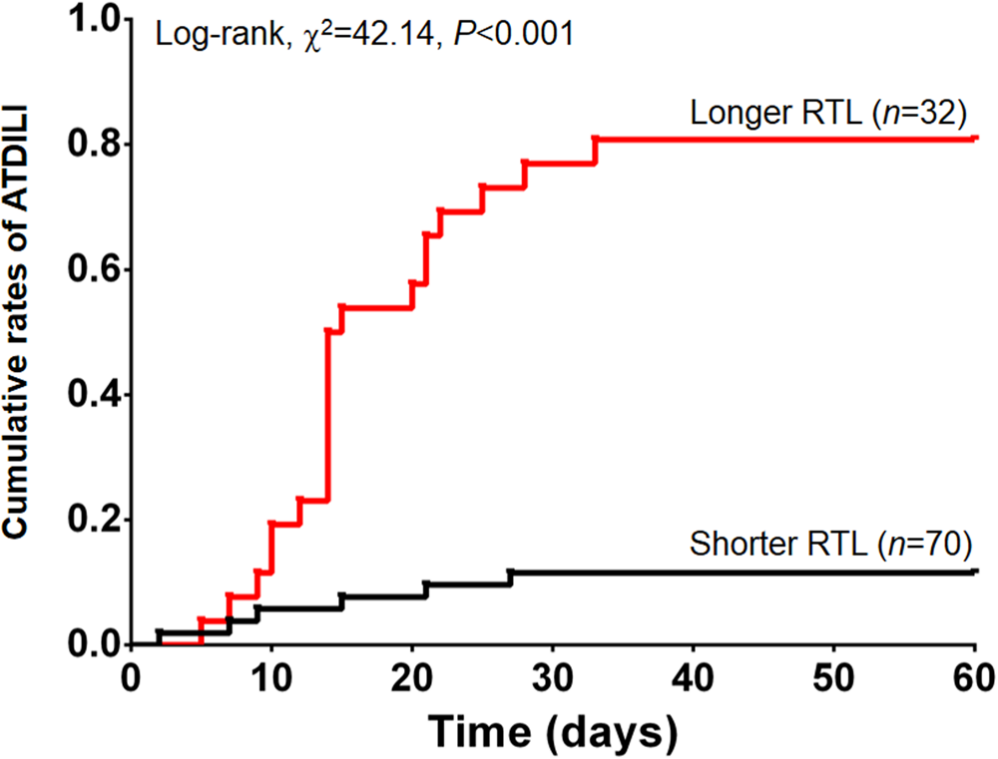
Kaplan-Meier curve for occurrence of ATDILI. Kaplan-Meier curve represents cumulative rates of ATDILI between tuberculosis patients with longer RTL and shorter RTL. Abbreviations: ATDILI, anti-tuberculosis drug-induced liver injury; RTL, relative telomere length.

Given longer RTL as an independent risk factor for ATDILI, we performed Kaplan-Meier analysis to investigate the effect of longer RTL on cumulative rates of ATDILI in tuberculosis patients. The analysis demonstrated that the cumulative rates of ATDILI were significantly higher in tuberculosis patients with longer RTL (80.77%) than that in those with shorter RTL (11.54%) (log-rank, χ^2^ = 42.14, *P* < 0.001), as depicted in Fig. 2.

### RTL as a diagnostic marker for early ATDILI progression

**Figure 3:**
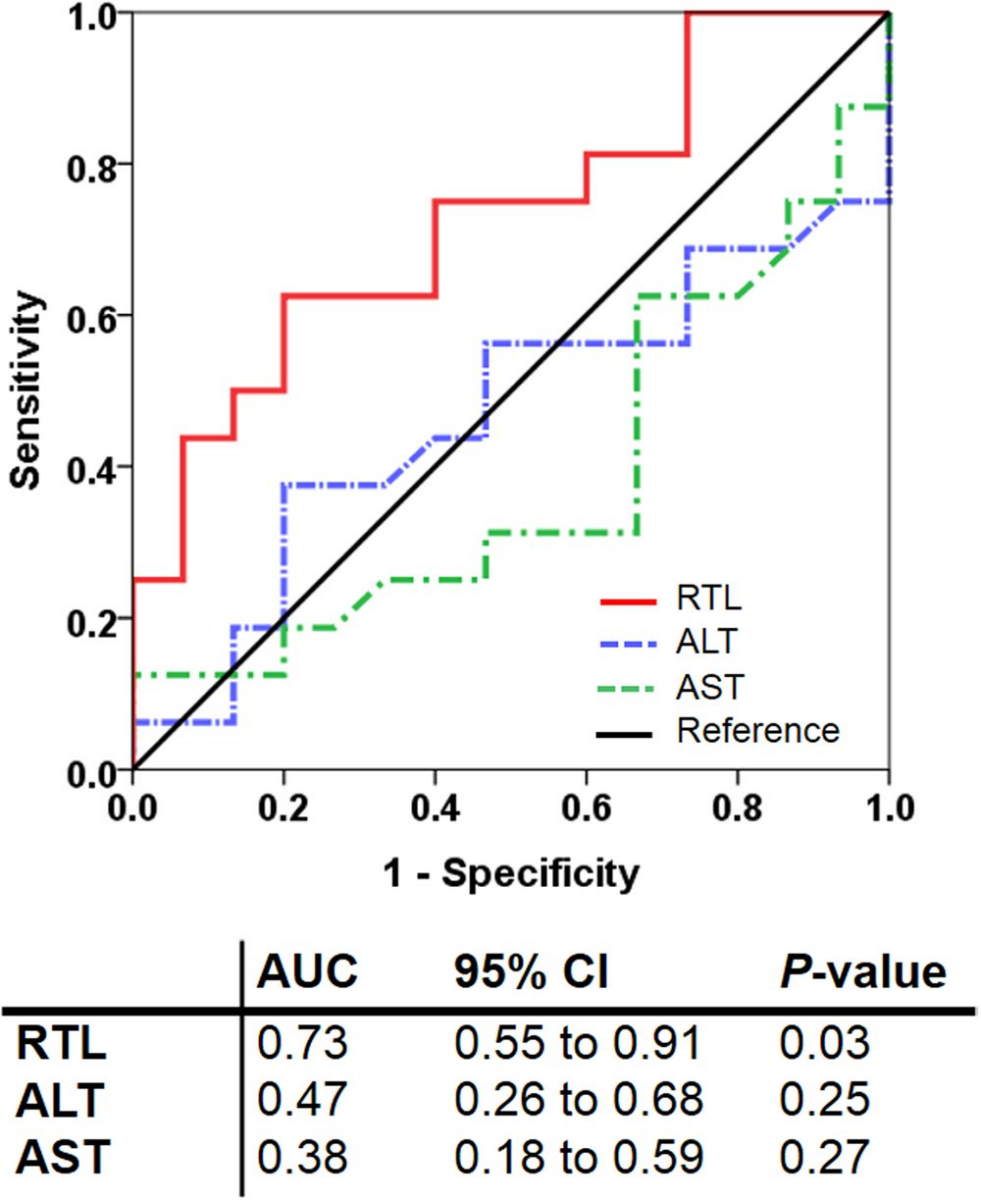
ROC revealing a diagnostic value of RTL as an ATDILI biomarker. Leukocyte RTL cloud be used as an early biomarker for distinguishing tuberculosis patients with ATDILI from non-ATDILI. Abbreviations: ATDILI, anti-tuberculosis drug-induced liver injury; ROC, receiver operating characteristic; RTL, relative telomere length.

To additionally identify RTL in blood leukocyte as an early indicator for the development and progression of ATDILI in tuberculosis patients, we calculated the area under curve (AUC) of the receiver operating characteristic (ROC), which was constructed using RTL values. The ROC curve analysis uncovered that the optimal cutoff value of RTL as a useful biomarker for discriminating tuberculosis patients with ATDILI from non-ATDILI was defined at 0.83, which yielded a sensitivity of 72.3%, a specificity of 60.0%, and an AUC of 0.73 (95% CI: 0.55 to 0.91; *P* = 0.03) (Fig. 3). On the other hand, the AUC of AST and ALT values measured within 7 days after treatment with the first-line anti-tuberculosis regimens was not statistically significant, suggesting that these traditional markers were not specific and selective for early ATDILI prediction.

## Discussion

Given that ageing has been implicated in the impaired clearance of drugs and their metabolites^16,17^, telomere length as an indicator for biological age of a cell are thought to be a potential reflector predisposition to age-associated diseases and could be used as a possible indicator of ATDILI progression in tuberculosis patients. To address these hypotheses, this is the first study to demonstrate a marked reduction in blood leukocyte RTL in tuberculosis patients with ATDILI compared to healthy controls. On the other hand, compared with the patients without ATDILI, RTL was significantly increased in those with ATDILI. Supporting this, our subsequent analysis revealed that tuberculosis patients with longer RTL had a considerably elevated risk of ATDILI. In particular, an elevation in RTL was found to be associated with increased serum levels of liver function parameters including AST and ALT in both the patients with and without ATDILI. All of our findings suggest that aberrant RTL in blood leukocyte may be associated with the developmental and progressive ATDILI in tuberculosis patients. Although the association between attrition of telomere length and ATDILI severity has not been investigated previously, there are considerable published data on RTL in a wide range of multifactorial diseases^10,18,19^. Amongst others, a case-control study by Savale *et al*.^10^ measured RTL in blood leukocyte of COPD patients and disclosed telomere shortening in those patients as compared with unaffected controls. This previous finding has been supported by a large cohort study conducted by Rode *et al*.^20^, which unveiled the associations of shorter RTL with decreased lung function and an increased risk of COPD in 46,396 individuals from the Danish general population. Apart from determination on RTL in COPD, a recent study by Udomsinprasert *et al*.^11^ investigated RTL in blood leukocyte of biliary atresia (BA) being chronic liver disease and reported that RTL was significantly reduced in BA patients, especially those with advanced-stage. This previous finding is consistent with a prior study, which noted telomere shortening in primary biliary cirrhosis patients with advanced-stage compared with healthy controls^21^. All above-mentioned findings led us to speculate that alterations in telomere length in blood leukocyte would reflect the impartment of hepatic function caused by stress and inflammation, and blood leukocyte RTL may have a diagnostic value as a novel biomarker indicating ATDILI progression in tuberculosis patients. With regard to a significantly close link between RTL and liver function parameters including AST and ALT values in tuberculosis patients, this study additionally explored the effect of longer RTL on the cumulative rates of hepatotoxicity induced by anti-tuberculosis agents and reported that tuberculosis patients with longer RTL exhibited remarkably increased rates of hepatotoxicity when compared with those with shorter RTL. In parallel with this result, the ROC curve analysis showed that sensitivity, specificity, and AUC values of RTL were greater than those of serum AST and ALT levels measured at 1–7 days after commencement of anti-tuberculosis treatment, suggesting the clinical utility of RTL as a diagnostic and prognostic marker for early phase of ATDILI progression. Although the role of telomere dysfunction in ATDILI remains poorly understood, the possible reason for reduced RTL in blood leukocyte of tuberculosis patients with ATDILI compared with healthy controls may be attributed to an increase in oxidative stress in hepatocytes characterized by the excessive production of reactive oxygen species (ROS) induced by anti-tuberculosis drugs, leading to cellular damage and eventually to telomere shortening in blood leukocyte. This hypothesis has been supported by an experimental study denoting that anti-tuberculosis drugs including rifampicin and isoniazid can cause increased ROS production-induce hepatocellular damage in mice^22^. Considering elevated RTL in the patients with ATDILI compared with those with non-ATDILI, it is tempting to assume that a marked increase in RTL in the patient with ATDILI may result from compensatory mechanisms by the body to fight against hepatic impairment or hepatocellular injury, which in turn induce regeneration process in hepatocytes and ultimately result in elongated telomere length. Taken together, these phenomena may help explain why shorter RTL was observed in tuberculosis patients with ATDILI compared to healthy controls, whereas longer RTL was found in the patients with ATDILI compared with those with non-ATDILI.

This investigation acknowledges certain caveats, which need to be taken into account. The most notable limitation is the fact that we did not determine RTL in tissue-specific liver cells of tuberculosis patients with ATDILI, due to ethical consideration. However, previous investigation showed a strongly positive association between RTL in blood leukocyte and RTL in the liver, implying that RTL in blood leukocyte would reflect alterations in RTL in the liver^11^. In support of this assumption, the inappropriate activation and homing of blood leukocyte to the microvasculature contribute to the pathological manifestations of liver diseases^23^, in which enhancing the production of several inflammatory mediators including pro-inflammatory cytokines and ROS caused by hepatocellular injury had been recognized as the pathologic event driving leukocyte extravasation into the hepatic parenchyma and the inflamed tissue mediated through triggering integrin activation and firm adhesion^24^. Furthermore, owing to the cross-sectional design, we were unable to drawn unequivocal conclusions on the causal relationships between aberrant RTL and ATDILI development. It is recommend that prospective cohorts with larger sample sizes will help to verify any relationships. Besides, primarily due to lack of data on biochemical parameters in healthy controls, determining the association between those factors and RTL in healthy volunteers was unachievable. Additionally, since the study participants are from hospital-based participants rather than the general population, there might be some risk of selection bias if they had any differences in terms of the studied exposures.

In summary, this is the first study to provide evidence showing that RTL in blood leukocyte of tuberculosis patients with ATDILI was significantly lower than that of age- and gender-matched healthy controls. When compared with the patients with non-ATDILI, RTL was found to be significantly increased in tuberculosis patients with ATDILI. Correspondingly, tuberculosis patients with longer RTL showed substantially an increased risk of ATDILI. Interestingly, subsequent analysis unveiled a close link between RTL and serum levels of AST and ALT. Besides a significant involvement of longer RTL in raised levels of liver function enzymes, longer RTL cloud predict the occurrence of hepatotoxicity in tuberculosis patients during therapy. Our findings suggest that RTL appears to have potential as an early biomarker indicating the development and progression of ATDILI. Further investigations are still essential for better understanding the precise role of telomeres in the pathogenesis of ATDILI, which may allow clinicians to develop strategies to reduce the occurrence of hepatotoxicity in tuberculosis patients and its adverse outcomes.

## Materials and methods

The study protocol conducted in conformity with the guidelines of the declaration of Helsinki was approved by the Institutional Review Board of the Faculty of Dentistry/Faculty of Pharmacy, Mahidol University (IRB number 2018/061.1110). All participants were fully informed regarding the study protocols and procedures prior to their entering the study. Written informed consent was obtained from the participants.

### Study participants

This case-control study consisted of 100 tuberculosis patients who were diagnosed by clinical blood test, a simple skin test, as well as histological findings and 100 age- and gender-matched unaffected volunteers who have no history of tuberculosis, autoimmune, or liver diseases. All tuberculosis patients received standard anti-tuberculosis regimens including isoniazid, rifampicin, pyrazinamide, as well as ethambutol, and the patients were classified into those with and without ATDILI groups, with regards to their blood levels of liver function parameters including AST, ALT, and TB. According to clinical practice guideline for tuberculosis treatment in Thailand^25^, tuberculosis patients with ATDILI had clinical features, as follows: (i) AST and/or ALT levels higher than three times upper limit of normal (ULN) along with one symptom of hepatitis including anorexia, fatigue, nausea vomiting, jaundice, liver enlargement and/or dark urine or (ii) AST and ALT levels higher than five times ULN or TB levels higher than three times ULN with or without symptom of hepatitis. None of the participants had underlying diseases such as hepatitis virus infection, liver cirrhosis, or previous history of liver diseases and metabolic syndromes. Besides, the patients who were treated with some drugs-induced liver injury consisting of methotrexate, phenytoin, phenobarbitone, carbamazepine, valproate, atenolol, labetalol, salicylates, allopurinol, quinine, quinidine, fluconazole, cimetidine, ethionamide, verapamil, probenecid, and halothane were excluded.

Venous blood samples drawn from tuberculosis patients within 7 days after initiation of anti-tuberculosis treatment and age- and gender matched healthy controls were collected in ethylenediaminetetraacetic acid tube to facilitate isolation of plasma as well as leukocytes and were then stored at −20 °C till utilized. Liver function parameters including AST, ALT, ALP, TB, and DB were routinely measured using an automated machine.

### Quantitative real-time polymerase chain reaction (PCR)

Genomic DNA was extracted from peripheral blood leukocytes using the QIAamp DNA Blood Mini Kit (Qiagen, CA, USA), according to the manufacturer’s protocol. Relative telomere length (RTL) was determined by quantitative real-time PCR, as described previously^26^. Briefly, PCRs were performed using Mx3000P Real-Time PCR System (Agilent, Santa Clara, CA, USA) with QPCR Green Master Mix HRox (biotechrabbit GmbH, Hennigsdorf, Germany). The primers used for the telomere repeat copy number and the single-copy gene copy number amplification were, as follows: telomere forward 5′-CGGTTTGTTTGGGTTTGGGTTTGGGTTTGGGTTTGGGTT-3′; telomere reverse 5′-GGCTTGCCTTACCCTTACCCTTACCCTTACCCTTACCCT-3′; single-copy gene forward 5′-CAGCAAGTGGGAAGGTGTAATCC-3′; and, single-copy gene reverse 5′-CCCATTCTATCATCAACGGGTACAA-3′. RTL was measured according to the ratio of the telomere repeat copy number (T) to the single-copy gene copy number (S) in each given sample. In each sample, the quantity of telomere repeats and the quantity of single-copy genes were normalized to a reference DNA sample (from a single individual)^11^.

### Statistical analysis

All statistical analyses were performed using the statistical package for social sciences version 22.0 (SPSS, Inc., Chicago, IL, USA). Demographic and clinical characteristics between groups were evaluated using Chi-square tests and Student’s unpaired *t*-test where appropriate. Comparisons among each group were executed by Mann-Whitney *U* test (for 2 groups) or Kruskal-Wallis *H* test (for >2 groups). Unconditional logistic regression models were utilized to identify risk factors for ATDILI. Correlations were analyzed by Spearman’s rho correlation, and multivariate logistic regression models were conducted to determine the roles of confounding factors. Receiver operating characteristic (ROC) curve and the area under the ROC curve (AUC) were calculated to estimate the feasible use of RTL as a potential marker for ATDILI progression. The Kaplan-Meier curves were drawn, with end points of ATDILI occurrence, in which the differences of curves were determined using log-rank test. Data are represented as mean ± standard deviation (SD). For all analyses, a two-tailed *P*-value of less than 0.05 was considered to be statistically significant.

## Supplementary information

Supplementary Information.
