# Supplementary material for: Leukocyte telomere length as a diagnostic biomarker for anti-tuberculosis drug-induced liver injury
Source: Sci Rep. 2020 Mar 27;10:5628. doi: 10.1038/s41598-020-62635-2 (PMC7101399; doi:10.1038/s41598-020-62635-2)
Supplement: Supplementary file 1 — Supplementary Information. [file 41598_2020_62635_MOESM1_ESM.docx]

**Leukocyte telomere length as a diagnostic biomarker for anti-tuberculosis drug-induced liver injury**

Wanvisa Udomsinprasert^1^, Noppadol Chanhom^1^, Supharat Suvichapanich^1^, Sukanya Wattanapokayakit^2^, Surakameth Mahasirimongkol^2^, Wasun Chantratita^3^, Jiraphun Jittikoon^1^

**Supplementary table 1** Logistic regression analysis for identifying risk factors of ATDILI.

| **Variables** | **Tuberculosis patients** | | **OR (95%CI)** | ***P* value** |
| --- | --- | --- | --- | --- |
|  | **ATDILI** | **Non-ATDILI** |  |  |
| Number | 49 | 53 |  |  |
| Age (years) |  |  |  |  |
| <50 years | 25 (51.0%) | 29 (54.7%) | 1 |  |
| ≥50 years | 24 (49.0%) | 23 (45.3%) | 0.83 (0.38 to 1.81) | 0.63 |
| Gender |  |  |  |  |
| Female | 21 (42.9%) | 16 (30.2%) | 1 |  |
| Male | 28 (57.1%) | 37 (69.8%) | 1.73 (0.80 to 4.13) | 0.19 |
| BMI (kg/m^2^) |  |  |  |  |
| <25 kg/m^2^ | 38 (77.6%) | 41 (77.4%) | 1 |  |
| ≥25 kg/m^2^ | 11 (22.4%) | 12 (22.6%) | 1.01 (0.40 to 2.56) | 0.98 |
| Drinking status |  |  |  |  |
| Never | 34 (69.4%) | 32 (60.4%) | 1 |  |
| Ever | 15 (30.6%) | 21 (39.6%) | 1.49 (0.66 to 3.38) | 0.34 |
| Smoking status |  |  |  |  |
| Never | 34 (69.4%) | 32 (60.4%) | 1 |  |
| Ever | 15 (30.6%) | 21 (39.6%) | 1.49 (0.66 to 3.38) | 0.34 |

Abbreviations: ATDILI, anti-tuberculosis-induced liver injury; BMI, body mass index; CI, confident interval; OR, odds ratio; RTL, relative telomere length.
